# Global incidence and prevalence in uveal melanoma

**DOI:** 10.1016/j.aopr.2024.10.001

**Published:** 2024-10-09

**Authors:** Xincen Hou, Alexander C. Rokohl, Xueting Li, Yongwei Guo, Xiaojun Ju, Wanlin Fan, Ludwig M. Heindl

**Affiliations:** a Department of Ophthalmology, University of Cologne, Faculty of Medicine and University Hospital Cologne, Cologne, Germany; b Center for Integrated Oncology (CIO), Aachen-Bonn-Cologne-Duesseldorf, Cologne, Germany; c Eye Center, Second Affiliated Hospital of Zhejiang University School of Medicine, Hangzhou, China

**Keywords:** Uveal melanoma, Incidence, Epidemiology

## Abstract

**Purpose:**

The most common intraocular cancer in adults is uveal melanoma (UM). This study aimed to investigate and report the incidence and prognosis of UM in different regions of the world.

**Methods:**

We retrieved relevant data on UM from the PubMed database and analyzed its global incidence and prognosis. All data was obtained from a national population-based registry, with publication dates ranging from 2013 to 2023.

**Results:**

The incidence rates of UM vary across different regions: in the United States, rates were 5.1 per million (1993–2008) and 5.2 per million (1973–2013); in Canada, rates ranged from 3.34 per million (1992–2010) to 5.09 per million (2011–2017); in Republic of Korea, the rate was 0.42 per million (1999–2011); in New Zealand, it was 5.56 per million (2000–2020); in Australia, it was 7.6 per million (1982–2014); and in Europe, rates ranged from 3.1 to 5.8 per million (1995–2002). Among European countries, Sweden (5.6 per million (1960–2009)), Germany (6.41 per million (2009–2015)), Poland (6.67 per million (2010–2017)), and the United Kingdom (10 per million (1999–2010)).

**Conclusions:**

The most common site of occurrence for UM is in the choroid. Limited data suggest a stable trend in UM incidence rates across the included countries, but significant differences in incidence rates exist among different countries and regions, with notably lower rates in Asian countries compared to Europe, North America, and Oceania. In general, the incidence rate in males is slightly higher compared to that in females.

## 1 Introduction

Intraocular tumors can be divided into benign and malignant tumors. Benign tumors primarily include astrocytomas, choroidal nevus, choroidal osteoma, choroidal hemangioma, and retinal capillary hemangioma. Intraocular malignant tumors mainly include retinoblastoma, medulloepithelioma, uveal melanoma, intraocular lymphoma, and metastasis. Retinoblastoma is the most common intraocular cancer in children, and uveal melanoma is the most common primary intraocular cancer in adults.^1^^,^^2^ They are relatively rare but serious blinding and even life-threatening diseases for patients.

Uveal melanoma (UM) is the most common primary intraocular malignant tumor in adults, accounting for 79%–81% of ocular melanomas and 3%–5% of all melanomas, with 5% originating from the iris, 10% from the ciliary body, and the majority (85%) from the choroid.^3^, ^4^, ^5^, ^6^ The global average incidence of iris melanoma is 1–9 cases per million, with a better prognosis and lower mortality rate compared to choroidal melanoma. There are significant geographic and racial variations, predominantly affecting Caucasians, followed by East Asians, with fewer occurrences among individuals of African descent.^1^^,^^7^ Risk factors include fair skin, light iris color, ancestry from northern latitudes, and ocular/ocular dermal melanocytosis.^1^. UM has a high rate of metastasis and mortality. Common sites of metastasis include the liver (89%), lungs (29%), and bones (17%)^3^^,^^8^^,^^9^ Once a patient develops metastasis, it means shorter survival and poorer prognosis.

This article aims to provide a comprehensive overview of the contemporary epidemiology of uveal melanoma, focusing on population-based incidence, prevalence characteristics, and prognosis across diverse regions worldwide.

## 2 Methods

We retrieved relevant data on UM from the PubMed database and analyzed its global incidence and prognosis. We used the following MeSH terms to search for literature: "mortality OR survival" AND "incidence" AND "uveal melanoma OR choroidal melanoma OR iris melanoma OR ciliary body melanoma". The inclusion criteria were defined as follows: data sourced from a national population-based registry; malignant tumors located in the uvea; the publication time of the article is between 1993 and 2023. Excluding small-scale studies such as those conducted in hospitals, local areas, and communities. Studies in which the language of the article was not English, the type of article was a case report, only crude incidence rates were calculated, and the lack of full text was also excluded. Descriptive statistical analyses of these studies were performed after the selection was completed and graphed using GraphPad Prism 9.5.1 (GraphPad Software).

## 3 Results

Using MeSH terms searches, a total of 97 articles were retrieved from PubMed. After our screening process, 25 articles on the incidence and prognosis of UM were included (Table 1, Table 2). We measure the incidence rate of uveal melanoma using age-standardized incidence rates(ASR). We found that the highest incidence rate was in the UK region, with a rate of 10.0 (95% CI: NA) per million, and the lowest was in Republic of Korea, with a rate of 0.42 (95% CI: 0.38–0.47) per million.In the United States, rates were 5.1 per million (1993–2008) and 5.2 per million (1973–2013); in Canada, rates ranged from 3.34 per million (1992–2010) to 5.09 per million (2011–2017); in Republic of Korea, the rate was 0.42 per million (1999–2011); in New Zealand, it was 5.56 per million (2000–2020); in Australia, it was 7.6 per million (1982–2014); and in Europe, rates ranged from 3.1 to 5.8 per million (1995–2002). Incidence rates were generally higher in the European region than in Oceania and North America, with the lowest rates in Asia. As for the prognosis of uveal melanoma, the global overall survival rates at 5 and 10 years ranged from 60.3% to 84% and 51%–71.4%, respectively, but some studies did not measure survival but rather mortality as an indicator, for example, in the statistics for Norway, the mortality rate was 3.5% (95% CI 3.1–3.9) per 1000 person-years of UM.

**Table 1:** Incidences studies on uveal Melanoma(UM).

Study group (Pub Year)	Country	Study size	Time period	Incidence rate per 1,000,000 person-years(95% CI)
Park (2015)	Republic of Korea	326	1999–2011	0.42 (0.38–0.47)
Singh (2011)	USA	4070	1973–2008	5.10 (4.8–5.3)
Aronow (2018)	USA	4999	1973–2013	5.20 (5.0–5.4)
Viktor (2022)	Sweden	3898	1960–2009	5.60–9.60 NA
Nowak (2022)	Poland	1632	2010–2017	6.67 NA
Conte (2023)	Canada	2215/1215	1992-2010/2011-2017	3.75/6.36
Aaron (2023)	Australia	4617	1982–2014	7.60 (7.3–7.9)
Alfaar (2022)	Germany	3654	2009–2015	6.41NA
Joevy (2023)	New Zealand	703	2000–2020	5.56 ​± ​1.02 NA
Mallone (2012)	Europe	4097	1995–2002	4.39 NA
Keenan (2012)	England	2171	1999–2010	10.0 NA
Isager (2005)	Denmark	388	1943–1997	Male7.8 NA/Female 6.5 NA

**Table 2:** Mortality/survival rate of UM.

Study group	Country	Study size	Time period	Prognosis
Tomas (2021)	USA	10678	1975–2016	Relative survival (RS) plateaued to 60% across 20–30 years. Excess absolute risk (EAR) parametric modeling yielded a survival probability of 57%.
Thaïs Tong (2023)	Netherlands	5036	1989–2019	The five- and ten-year overall survival between 1989 and 2004 were 61% and 46%, respectively. In the2005-2019, the five- and ten-year OS were 67% and 51%.
Ahmad Alfaar (2022)	Germany	3654	2009–2015	The 5-year overall survival stood at 47%, while the cancer-specific survival stood at 84%.
Nowak (2022)	Poland	1632	2010–2017	The 5-year overall survival was 60.76%.
Aaron Jamison (2019)	Scotland	218	1998–2002	15-year melanoma-specific survival of 83.8%, 81.3% and 100% for choroidal, ciliary body and iris melanomas, respectively.
San Jun Park (2017)	Republic of Korea	344	1999–2012	The observed 5-year survival probability from all-cause death was 75%.
Avenell L Chew (2015)	Western Australia	308	1981–2005	Relative survival rates for the entire cohort were 88.2%, 81.4% and 71.4% at 3, 5 and 10 years, respectively.
Trude E (2023)	Norway	960	1990–2017	Mortality per 1000 person-years was 3.5 (95% CI 3.1–3.9) for UM and 3.0 (2.6–3.4) for other causes.
Louise (2003)	Sweden	2997	1960–1998	The 5-year crude survival rate was 60.3% and the relative survival 70.1%. After 10 years, the rates were 42.5% and 59.4%, respectively.
Mallone (2012)	Europe	4103	1995–2002	Five-year relative survival rates (%):68.9%
Burr (2007)	England and Wales	4308	1986–2001	Relative survival from uveal melanoma was 95% at 1 year and 72% at 5 years.
Isager (2006)	Denmark	2319	1943–1997	The 5-year relative survival remained stable for the choroid/ciliary body at 66% for men and 69% for women, while for the iris, it was 90% for men and 99% for women.The 10-year relative survival remained stable for the choroid/ciliary body at 55% for men and 57% for women, while for the iris, it was 85% for men and 101% for women.
Frenkel (2009)	Israel	558	1988–2007	The 5-year melanoma-related mortality rate was 11.4%, the 10-year rate was 17.0%, and the 15-year rate was 23.3%.

## 4 Discussion

### 4.1 Incidence of uveal melanoma in various parts of the world

#### 4.1.1 North America

##### 4.1.1.1 America(1973–2008/1973-2013)

**Fig. 1:**
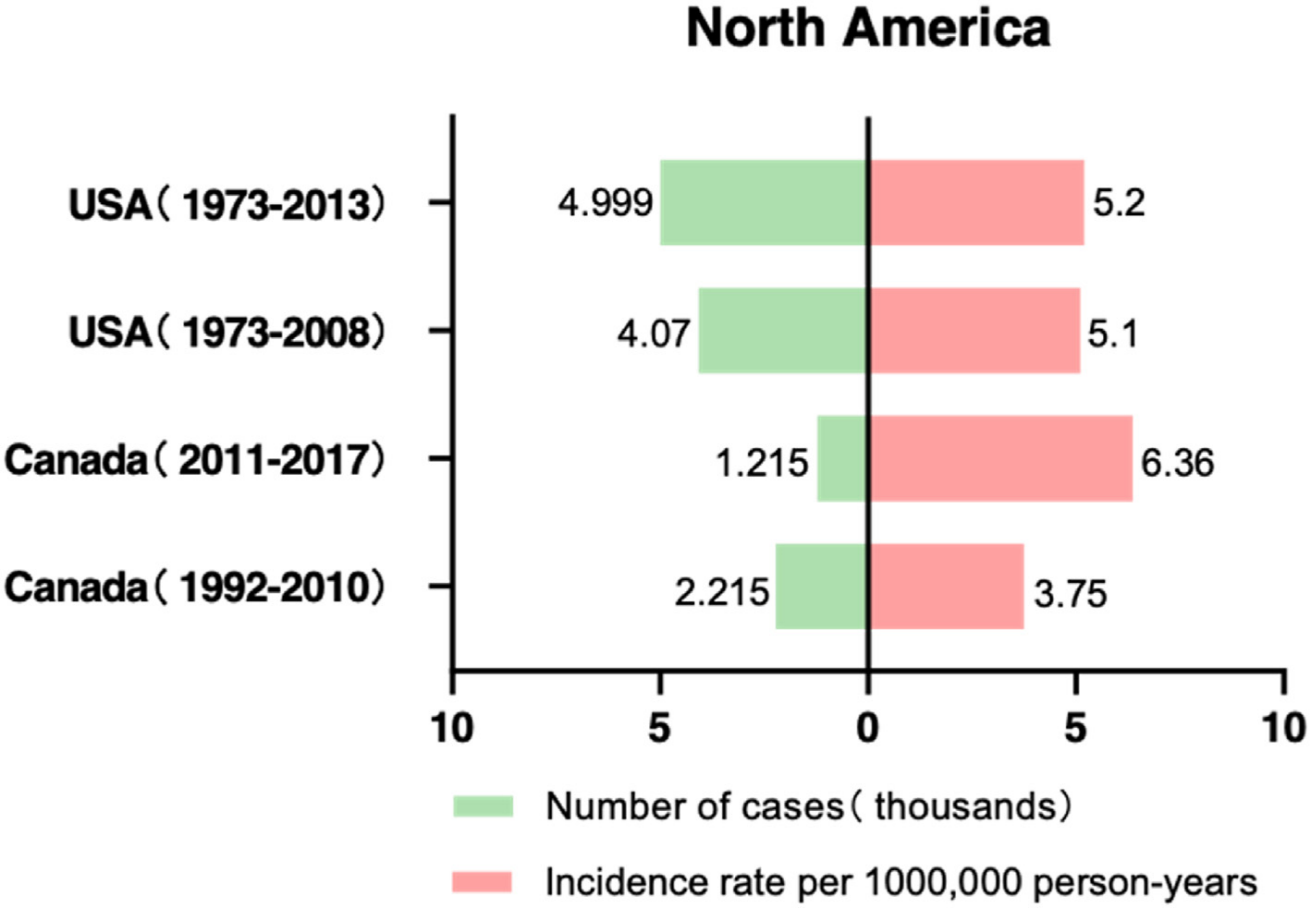
Number and prevalence of UM in North America.

**Fig. 2:**
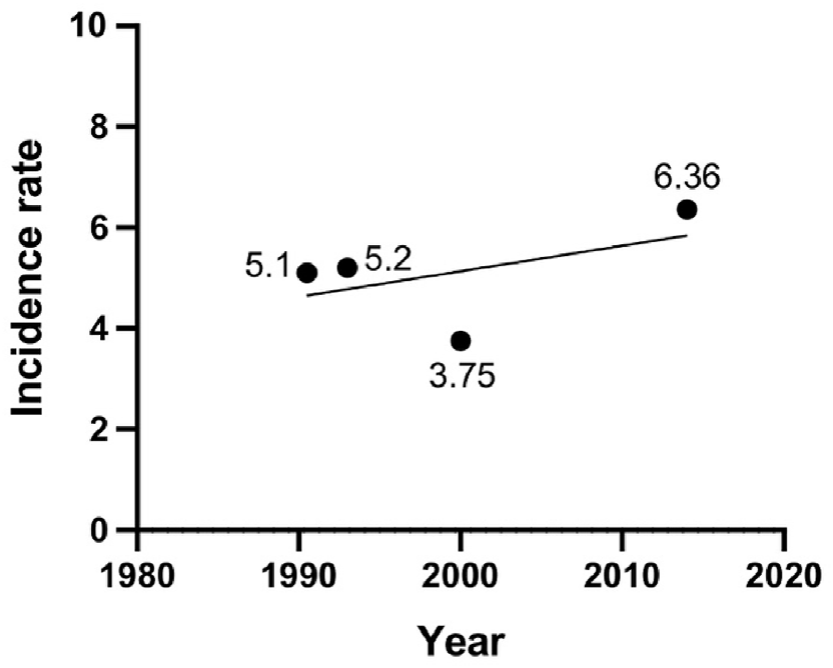
Incidence trends of UM in North America.

Singh, A. D. et al.^3^ used the Surveillance, Epidemiology, and End Results (SEER) Program database, which is one of the most authoritative sources of cancer-related statistics in the United States, to estimate incidence rates between 1973 and 2008, including a total of 4070 patients. The average ASR of UM remained stable during the study period at 5.1 cases per million population**.** The incidence rate also did not change significantly in recent years from 1997 to 2008 (5.1, 95% CI: 4.7–5.4) compared with the series with SEER data from 1973 to 1997 (5.0, 95% CI: 4.7–5.4).^10^ Although the overall age-adjusted incidence rate of UM remained stable between 1973 and 2008, there were significant differences between sexes (men: 5.8, 95% CI: 5.5–6.2; women: 4.4, 95% CI: 4.2–4.7). A study conducted by Aronow, M. E. and colleagues^11^ utilized the SEER database to explore the incidence of UM from 1973 to 2013. The overall ASR was 5.2 cases per million person-years. Predominantly, the affected patients were white (98%), and there was no significant difference in gender distribution, with 52.3% males and 47.7% females. However, the ASR was notably higher among males, with 6.0 cases per million population, compared to 4.5 cases among females. These findings are consistent with those observed from 1973 to 2008, suggesting a stable incidence of UM in the United States over the past 30 years (Fig. 1, Fig. 2).

##### 4.1.1.2 Canada(2011–2017)

In Canada, Conte, S. et al.^12^ analyzed the epidemiology of UM from 2011 to 2017, extracting 1215 cases from the Canadian Cancer Registry and comparing them with data from 1992 to 2010.^13^ The results revealed that the ASIR of UM during 1992–2010 was 3.75 per million person-years (3.93 for males and 3.54 for females). Compared with the ASR of 6.36 per million person-years during 2011–2017 (6.50 for males and 6.22 for females), the incidence of UM has significantly increased during 2011–2017, with a slightly faster growth rate in females than in males. The most common anatomical location of the lesions was the choroid, accounting for 84.2% and 89.3% in the two respective studies, while the iris, ciliary body, lens, and sclera remained the second most common sites (Fig. 1, Fig. 2).

#### 4.1.2 Asia

##### 4.1.2.1 Republic of Korea(1999–2011)

**Fig. 3:**
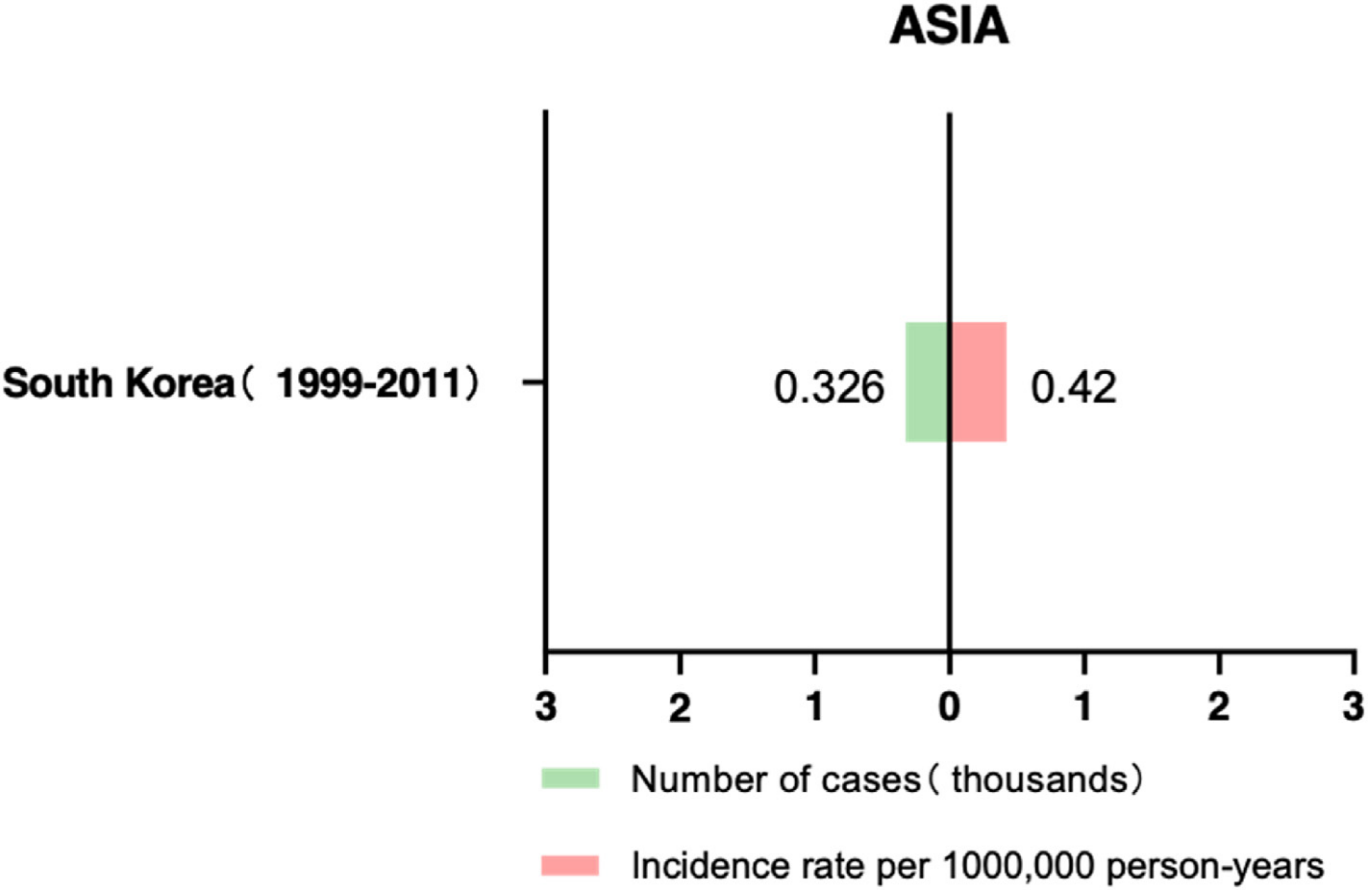
Number and prevalence of UM in Asia.

In 2015, a study conducted by Park, S. J. et al.^14^ reported the epidemiological characteristics and temporal trends of UM from 1999 to 2011. Based on information collected from the Korea Central Cancer Registry, the research found that the ASR of UM was 0.42 per million person-years over 12 years. The ASIR of UM diagnosed from 2006 to 2011 was higher than that diagnosed from 1999 to 2005, showing an increasing trend. This might be attributed to the increasing screening examinations in Korea during the study period, which could have influenced the rise in the incidence of choroidal melanoma. Additionally, no significant difference in the incidence rate between males and females was observed in this study. This could be due to inherent differences in uveal melanoma characteristics between Asian populations and Caucasian populations, or it could be because the number of cases was too small to reveal any differences (Fig. 3).

#### 4.1.3 Oceania

##### 4.1.3.1 Australia(1982–2014)

**Fig. 4:**
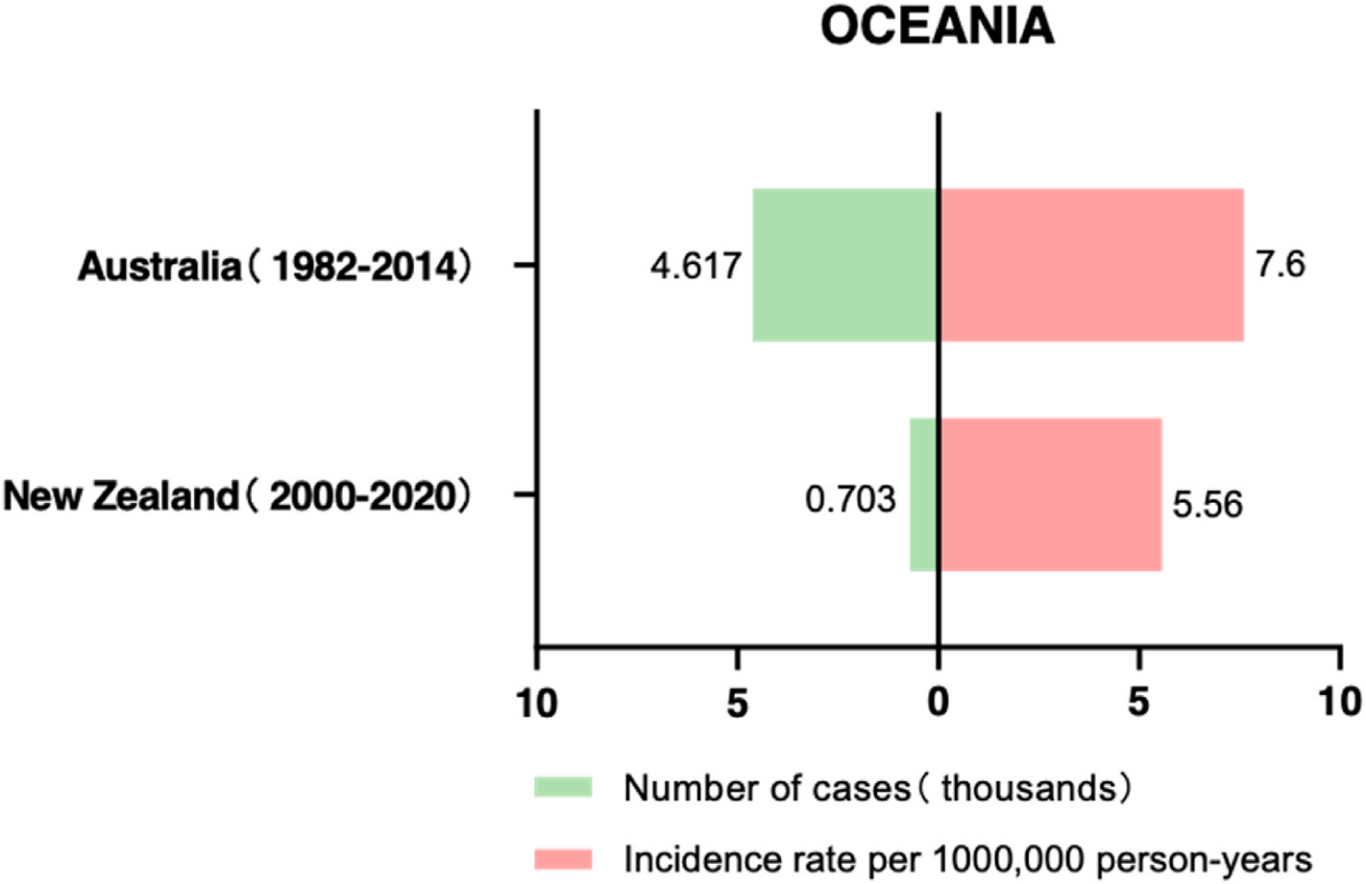
Number and prevalence of UM in Oceania.

Beasley et al. reported the incidence and clinical characteristics of uveal melanoma (UM) in the Australian population.^15^ Among melanomas occurring from 1982 to 2014, 4617 cases were classified as UM. Of these, 3230 (70%) were classified as choroidal and 577 as iris or ciliary body (12.5%). The average age-standardized rate (ASR) of UM was 7.6 per million persons (95% CI 7.3 to 7.9) (Fig. 4).The age-standardized incidence rate remained relatively stable overall during this period. Compared to females, males had a higher ASR, with rates of 6.9 and 8.4 per million persons, respectively. Previous studies have suggested that sun exposure, welding ultraviolet radiation, or certain specific occupational exposures to carcinogens are associated with an increased risk of UM, and males are more likely to be exposed to chemical and sunlight carcinogens,^16^, ^17^, ^18^, ^19^, which may partially explain the differences in incidence rates between genders.

##### 4.1.3.2 New Zealand(2000–2020)

In 2023, a New Zealand study reported epidemiological data on UM from 2000 to 2020. The study extracted data from 703 patients from the New Zealand Cancer Registry.^20^ (Fig. 4). UM was found to be evenly distributed between gender and the laterality of eyes, with the highest incidence among Europeans (95%), followed by Māori (4%), and Pacific and Asian <1% each. Overall, the choroid was the most common primary site (76%), followed by the ciliary body and iris (18%), and 6% originated from nonspecific or other sites. The region's annual ASIR per million New Zealand population in 2000–2004, 2005–2009, 2010–2014, and 2015–2020 were 5.57, 5.91, 6.16, and 4.75 respectively, corresponding to current global estimates for the European population.^11^^,^^21^, ^22^, ^23^

#### 4.1.4 Europe

Mallone et al.^24^ conducted a study on the incidence of UM throughout Europe during 1995–2002 and found that the overall incidence rate in Europe was 4.4 cases per million people, ranging from 3.1 in southern Europe to 5.8 in northern Europe. The reason for this phenomenon may be that the incidence of UM is related to latitude, with people living at different latitudes having different eye colors. People living in southern Europe usually have darker eye color than those in northern Europe.^25^

**Fig. 5:**
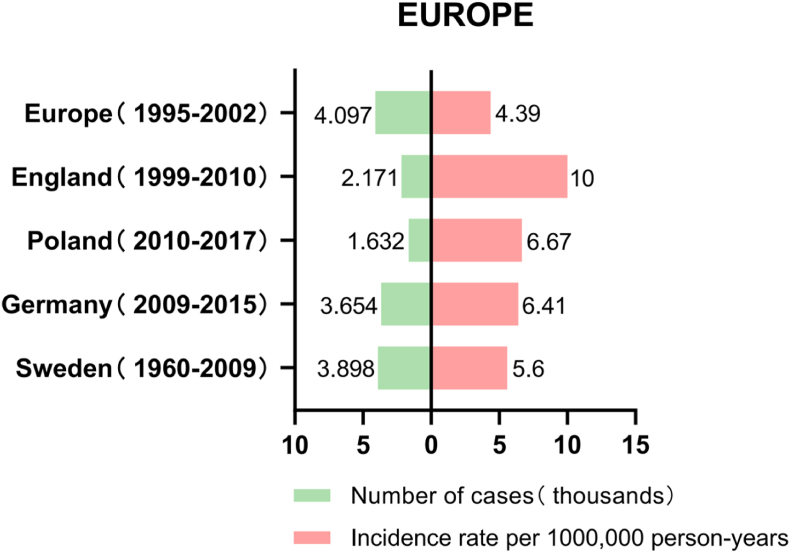
Number and prevalence of UM in Europe.

**Fig. 6:**
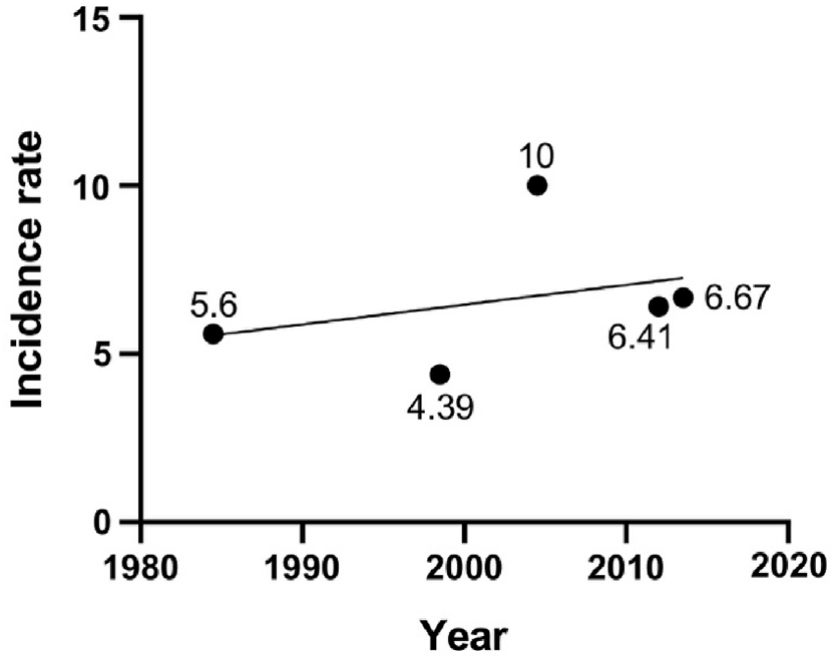
Incidence trends of UM in Europe.

In Sweden, a study during 1960–2009 showed an annual incidence of this cancer of 5.6 cases per million people.^25^ A study conducted in the United Kingdom between 1999 and 2010 found an annual incidence rate of 10 cases per million people for this type of cancer.^26^ The incidence rates were similar between the two study periods, from 2001 to 2005 and from 2006 to 2010. In both males and females, the incidence rates generally increased with age, with the highest rates observed in the 70–79 age group. Among individuals aged 50 and above, males consistently had higher incidence rates than females. In a survey in Germany between 2009 and 2015, the annual incidence rate of UM was found to be 6.41 cases per million,^27^ and a study in Poland from 2010 to 2017 had a similar incidence rate of 6.67 cases per million.^28^ (Fig. 5, Fig. 6)

In addition, the incidence rates of men and women are slightly different. A study in Denmark found that the incidence rate per million for men was 7.8, whereas for women it was 6.5,^29^ and similar results were found in a survey of studies in Europe, where the incidence rates per million were 4.94 and 3.97 for men and women, respectively.^24^ Furthermore, in studies conducted in Germany, the United States, and Northern Ireland, it was found that the incidence rate of uveal melanoma is higher in males than in females.^3^^,^^11^^,^^27^^,^^30^

### 4.2 Survival status of uveal melanoma in various parts of the world

#### 4.2.1 North America

In the United States, a study spanning from 1975 to 2016 indicated a stable relative survival (RS) of approximately 60% over a 20–30 year period for UM. By the end of 2016, more than half of the patients remained alive. The Kaplan-Meier estimates for metastasis-free survival at 10, 20, and 30 years were 0.729, 0.648, and 0.616, respectively. The cumulative probabilities of death due to metastatic melanoma were 0.241 ​at 10 years, 0.289 ​at 20 years, and 0.301 ​at 30 years. During the first five years following diagnosis, deaths attributable to uveal melanoma accounted for 1.3%, with a marked decline after the 10-year mark.^31^

#### 4.2.2 Europe

Moreover, In Europe, a study covering 1995–2002 reported a 5-year relative mortality rate of 68.9%.^24^ A study conducted in the Netherlands between 1989 and 2019 found an improved overall survival (OS). Between 1989 and 2004, the 5-year and 10-year survival rates were 61% and 46%, respectively. From 2005 to 2019, these rates rose to 67% and 51%, respectively. The investigation also highlighted that female gender and radiotherapy treatment correlated with improved OS.^32^ In Germany, a survey on UM survival rates from 2009 to 2015 showed a 5-year OS of 47% and a cancer-specific survival of 84%. This study also indicated that females and younger individuals exhibited higher overall survival rates.^27^ Nordic countries revealed varying UM death rates, with Norway's study from 1990 to 2017 indicating 3.5 per 1000 person-years (95% CI 3.1–3.9). In Sweden, a study found a 5-year crude survival rate of 60.3% and a relative survival of 70.1%, while the rates after 10 years were 42.5% and 59.4%, respectively.^33^^,^^34^

During the period from 1986 to 2001, a study in England and Wales revealed a one-year relative survival rate of 95% and a five-year relative survival rate of 72% for uveal melanoma. Notably, advanced age correlated with a poorer prognosis, but gender exhibited no such correlation.^35^ In Scotland between 1998 and 2002, a study delineated 15 years of melanoma-specific survival for different uveal parts. The iris exhibited the highest survival rate at 100%, while the rates for the choroid and ciliary body were 83.8% and 81.3%, respectively.^36^

#### 4.2.3 Oceania

A study in Western Australia spanning from 1981 to 2005 discovered relative survival rates for the entire cohort at 88.2%, 81.4%, and 71.4% at 3, 5, and 10 years, respectively. Factors associated with poorer survival included mixed-cell tumor morphology, tumor location in the ciliary body, and apical tumor height greater than 5 ​mm. Among patients who underwent enucleation, those diagnosed between 1998 and 2005 experienced twice the rate of mortality. For the 17 patients who developed metastasis, the median survival from the time of metastasis diagnosis was 3.1 months.^37^ These survival rates are consistent with those reported in the United States and are more favorable compared to most European-based studies.

#### 4.2.4 Asia

There are a few studies that have investigated the survival rates of uveal melanoma in Asia. A study conducted in Korea from 1999 to 2012 reported a 5-year OS of 75%.^38^ In an Israeli survey spanning from 1988 to 2007, the mortality rates associated with melanoma were determined to be 11.4% at 5 years, 17.0% at 10 years, and 23.3% at 15 years.^39^

Despite the diverse and rapidly evolving treatment methods for UM, including plaque radiotherapy and enucleation, and eye-preserving treatments like transpupillary thermotherapy (TTT), particle radiotherapy, stereotactic radiotherapy, and local excision of eye tumors,^40^^,^^41^ the long-term survival rate for uveal melanoma patients remains relatively low, with approximately 50% of UM patients eventually experiencing hematogenous metastasis, often involving the liver. Early detection and treatment are crucial for enhancing the survival rate of UM patients.^42^

The limitation of this study is that we attempted to obtain data from publicly available online cancer databases, such as the Global Cancer Observatory (GCO) database. Unfortunately, these databases only contain data on all ocular melanomas and do not provide specific records for uveal melanoma. Therefore, our data were obtained from published articles rather than from the original databases.

## 5 Conclusions

In summary, while limited data indicate a stable trend in UM incidence rates across the studied countries, substantial disparities exist among various countries and regions, particularly with lower rates observed in Asian countries in contrast to Europe, North America, and Oceania. The incidence rate tends to be slightly higher in males compared to females. The overall incidence trend is stable, while the survival rate of patients has not significantly increased.

## Author contributions

The authors confirm contribution to the paper as follows: Conception and design of study: XH; Data collection: ACR, XJ; Analysis and interpretation of results: XH, XL; Drafting the manuscript: XH, WF; All authors reviewed the results and approved the final version of the manuscript.

## Funding

The present study was supported by the State Scholarship Fund from 10.13039/501100004543China Scholarship Council (Nr. 202108370094 to X.H.).

## Declaration of competing interest

The authors declare that they have no known competing financial interests or personal relationships that could have appeared to influence the work reported in this paper.

## Abbreviations

UM: Uveal Melanoma
SEER: Surveillance, Epidemiology, and End Results
ASR: Age-standardized incidence rates
OS: Overall survival
RS: relative survival
